# Chronic Pulmonary Aspergillosis in a Patient With AIDS

**DOI:** 10.7759/cureus.14588

**Published:** 2021-04-20

**Authors:** Ranjan K Singh

**Affiliations:** 1 Internal Medicine, Anti-Retroviral Therapy Centre, District Hospital, Khagaria, IND

**Keywords:** aids, aspergillosis, hiv, itraconazole, chronic pulmonary aspergillosis

## Abstract

Chronic pulmonary aspergillosis (CPA) is caused by saprophytic fungi *Aspergillus* spp. Certain conditions predispose individuals to pulmonary aspergilloses, for example, neutropenia, prolonged steroid therapy, immunosuppressive drugs, and solid organ transplants. Individuals are infected with *Aspergillus *spores by inhalation. CPA is diagnosed through imaging features, such as cavities, fungal balls, peripheral air crescent signs, and the direct visualization of the *Aspergillus* spp. (microscopy or culture from biopsy) or immunological response to *Aspergillus* spp. (serum IgG confirms the diagnosis of CPA). All these should be present for at least three months.

An *Aspergillus* infection is uncommon in those with the human immunodeficiency virus (HIV) due to intact phagocytic cell function. However, HIV-infected individuals with CD4+ T cell < 100 cells/mL are more likely to experience disease progression. Chronic tubercular cavities predispose one to the colonization of cavities with *Aspergillus* spp. When HIV advances to AIDS (acquired immunodeficiency syndrome), the aspergilloma transforms into an invasive form.

## Introduction

*Aspergillus* spp. are saprophytic fungi, and their habitat is mostly found in soil and decaying vegetables. Intact respiratory tract epithelium, neutrophils, and Th1 cells (helper T cells) protect individuals from *Aspergillus* spp. Therefore, aspergillosis is uncommon despite that exposure to *Aspergillus* spp. is universal. Chronic pulmonary aspergillosis (CPA) is a progressive disease. Individuals are infected through inhalation. Certain conditions predispose individuals to pulmonary aspergillosis, for example, neutropenia, prolonged steroid therapy, immunosuppressive drugs, and solid organ transplants.

The consensus definition of CPA is chronic cavitary pulmonary aspergillosis. When it is left untreated, it leads to chronic fibrosing pulmonary aspergillosis in immunocompetent patients with prior or current lung disease. Other manifestations are *Aspergillus* nodule (<3 cm), solitary aspergilloma, and subacute invasive pulmonary aspergillosis (SIPA). The latter is a rapidly progressive disease in moderately immunocompromised patients. An overlap is often observed among these manifestations [1].

Aspergillosis is uncommon in human immunodeficiency virus (HIV) patients; there are a few cases of aspergillosis in this population despite HIV patients having severe immune suppression, i.e., AIDS (acquired immunodeficiency syndrome) (CD4+ T cell count < 200 cells/mL). For example, data show an overall incidence of CPA in HIV positive population to be 3.5 cases per 1000 persons-years [2].

In keeping with guidelines from the European Society for Clinical Microbiology and Infectious Diseases and others, CPA is confirmed through imaging features (e.g., one or more cavities, peripheral air crescent signs) and the direct visualization of *Aspergillus* spp. (microscopy or culture from biopsy) or immunological response to *Aspergillus* spp. (i.e., serum immunoglobulin G (IgG) confirms the diagnosis of CPA). All these changes should last for at least three months [1].

## Case presentation

A 24-year-old male HIV seropositive patient attended the Anti-Retroviral Therapy Centre since he was positive for HIV antibodies. He complained about an unresolved fever and cough with frequent spells of hemoptysis despite having anti-tuberculous treatments for the past 1.5 years. His previous chest X-ray showed fibrocavitary lesions in the lungs and had a sputum microscopy report positive for acid-fast bacillus (AFB). He was emaciated. The total leucocyte count was 11.4 x 10^9^/L, with a differential count (neutrophil) of 70%. Sputum microscopy and the nucleic acid amplification test for *Mycobacteria tuberculosis* came back negative.

**Figure 1:**
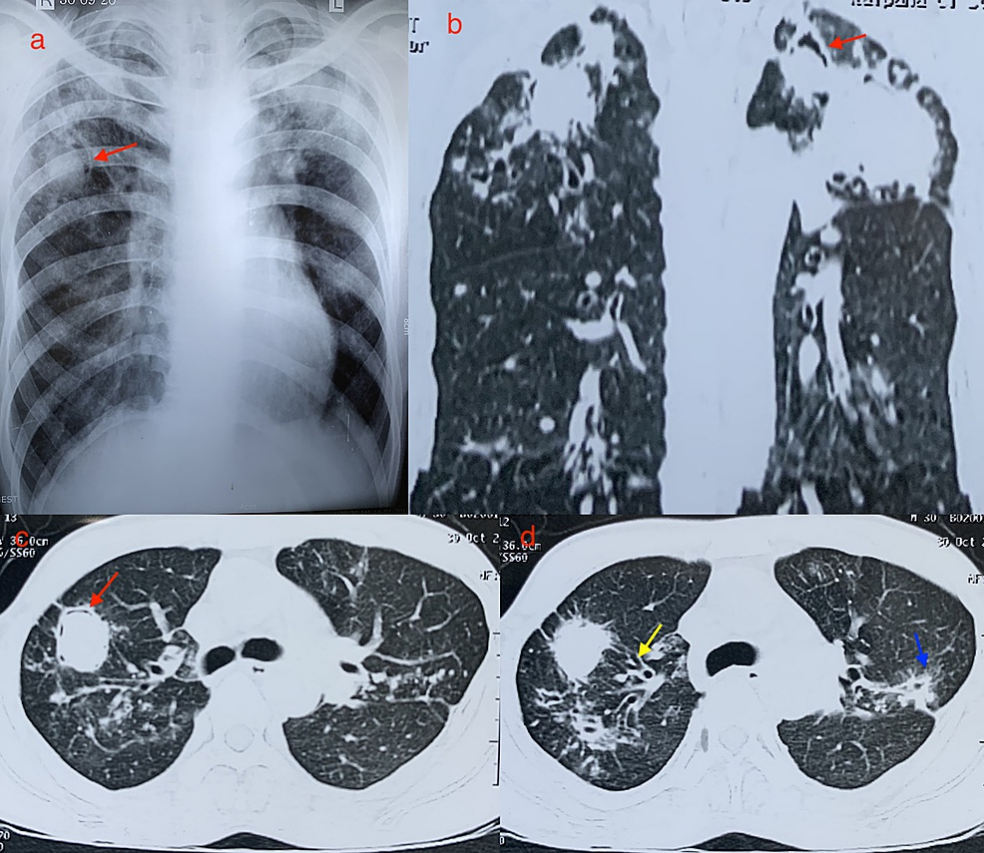
Chest X-ray and CT scan of the chest (sagittal and axial views). (a) Chest X-ray showing peripheral air crescent sign (red arrow). (b) Sagittal CT scan of the chest showing air crescent sign (red arrow). (c) Axial CT of the chest showing peripheral air crescent sign (red arrow). (d) Axial CT of the chest showing air crescent inside the nodule with a halo sign (blue arrow) and tree-in-bud (yellow arrow).

A chest X-ray (Figure 1a) and CT scan of the chest (Figures 1b, 1c) showed multiple fungal balls with a peripheral air crescent, as well as a tree-in-bud sign and a ‘halo’ sign (Figure 1d).

The CD4+ T cell count was 65 cells/mL. The anti-*Aspergillus* IgG in the serum was estimated at 6.5 units/mL (ref: positive > 1.2 units/mL).

Combined anti-retroviral drugs (tenofovir, lamivudine, and efavirenz) were started, along with the antifungal drug itraconazole 200 mg twice a day. After six months, the patient gained 6 kg and became asymptomatic. His CD4+ T cell count increased to 182 cells/mL.

## Discussion

An *Aspergillus *infection is uncommon among HIV patients due to their intact phagocytic cell functions. However, HIV-infected individuals with CD4+ T cell < 100 cells/mL are more likely to experience disease progression. Cavitary lesions in the lungs predispose one to the colonization of a cavity with *Aspergillus* spp. forming aspergilloma. As the HIV infection advanced with the decline of the CD4+ T cell count to 65 cells/mL, the aspergilloma transformed into an invasive form.

Although peripheral air crescent signs in chest X-ray and CT scans are characteristic of pulmonary aspergillosis, they can be seen in hydatid cyst, abscess, Wegener’s granuloma, and neoplasm. The tree-in-bud sign is also seen in the non-tuberculous mycobacteria-pulmonary disease (NTM-PD), endobronchial tuberculosis, cystic fibrosis, and rheumatoid arthritis [3]. A ‘halo’ sign in CT scan is defined as a macro-nodule surrounded by ground-glass opacity. The sign is an early indicator of invasive aspergillosis in a high-risk patient. It is also seen in other molds (*Zygomycetes*) and bacterial pathogens such as *Pseudomonas aeruginosa* [4]. Furthermore, *Aspergillus* IgG is a valuable biomarker for CPA diagnosis. The diagnostic sensitivity and specificity of *Aspergillus* spp. IgG estimated by enzyme-linked assay were 84.1% and 89.6%, respectively, in a prospective study carried out during 2016-2017 [5]. The presence of anti-*Aspergillus* antibodies differentiates infected and colonized patients with a 100% predictive value [1]. A negative result of *Aspergillus* IgG points to differentials such as NTM-PD, coccidioidomycosis, histoplasmosis, and pulmonary carcinoma [1].

The most common form of CPA, chronic cavitary pulmonary aspergillosis, has been best managed with long-term medical therapy using itraconazole or voriconazole. Majority of cases respond to the treatment by six months; however, the median duration of treatment was 46 weeks in an open-label prospective study [6]. Amphotericin-B has been tried in case of oral drug failure; however, therapeutic response is less optimal especially in HIV patients. Surgery is indicated for a patient having interactable hemoptysis with a solitary lesion in the lungs.

## Conclusions

CPA is uncommon in HIV patients as they have cell-mediated immune deficiencies with intact phagocytes. However, structural lung damage caused by prior infections can predispose one to an *Aspergillus* infection, which progresses rapidly in the face of immune suppression. Majority of patients respond to oral antifungal drugs along with anti-retroviral drugs.
